# The Overexpression of *Oryza sativa* L. *CYP85A1* Promotes Growth and Biomass Production in Transgenic Trees

**DOI:** 10.3390/ijms24076480

**Published:** 2023-03-30

**Authors:** Guodong Li, Xinzhuan Yao, Zhouzhuoer Chen, Xingyu Tian, Litang Lu

**Affiliations:** 1 The Key Laboratory of Plant Resources Conservation and Germplasm Innovation in Mountainous Region (Ministry of Education), College of Life Sciences/Institute of Agro-Bioengineering, Guizhou University, Guiyang 550025, China; 2 College of Tea Sciences, Institute of Plant Health & Medicine, Guizhou University, Guiyang 550025, China

**Keywords:** *Oryza sativa* L., *OsCYP85A1*, castasterone (CS), *Populus tomentosa*, biomass production, xylem

## Abstract

Brassinosteroids (BRs) are important hormones that play crucial roles in plant growth, reproduction, and responses to abiotic and biotic stresses. CYP85A1 is a castasterone (CS) synthase that catalyzes C-6 oxidation of 6-deoxocastasterone (6-deoxoCS) to CS, after which CS is converted into brassinolide (BL) in a reaction catalyzed by CYP85A2. Here, we report the functional characteristics of rice (*Oryza sativa* L.) *OsCYP85A1*. Constitutive expression of *OsCYP85A1* driven by the cauliflower mosaic virus 35S promoter increased endogenous BR levels and significantly promoted growth and biomass production in three groups of transgenic *Populus tomentosa* lines. The plant height and stem diameter of the transgenic poplar plants were increased by 17.6% and 33.6%, respectively, in comparison with control plants. Simultaneously, we showed that expression of *OsCYP85A1* enhanced xylem formation in transgenic poplar without affecting cell wall thickness or the composition of cellulose. Our findings suggest that *OsCYP85A1* represents a potential target candidate gene for engineering fast-growing trees with improved wood production.

## 1. Introduction

The class of compounds known as plant steroidal hormones contains more than 40 members, which are collectively designated brassinosteroids (BRs). Among BRs, castasterone (CS) and brassinolide (BL) are considered to have the most important biological roles [1,2]. BRs play critical roles in regulating plant developmental processes, including cell division, cell cycle, elongation, morphogenesis, reproduction, senescence, and stress-protective responses [3,4,5]. Simultaneously, BRs act as regulators of plant vascular structure formation, hypocotyl elongation, root growth, bud growth, xylem formation, and xylem differentiation [6,7,8].

In general, BL positively contributes to the cellular biochemical, physiological, and morphological processes of taller plants, especially cell division, antioxidant metabolism, gas exchange, and growth [6,9,10]. Previous reports suggest that CS activates BR signal transduction pathways with efficacy similar to that of BL. A rice lamina inclination bioassay revealed that CS induced lamina bending, although it was slightly less effective than BL [11]. CS is biosynthesized from 24-methylcholesterol via two parallel pathways, namely, the early and late C-6 oxidation pathways, after which CS is oxidized by 7-oxalactonation to produce BL. The rice CYP85A enzyme catalyzes C-6 oxidation of 6-deoxocastasterone (6-deoxoCS) to produce CS, a bioactive BR [11]. The tomato *Dwarf* gene encodes *CYP85A1*, which catalyzes the synthesis of CS from 6-deoxoCS [12]. Catabolism of BRs is another important factor that regulates their endogenous levels. In Arabidopsis, the inactivation of BRs is also catalyzed by P450 enzymes, among which the key genes are *CYP734A1* and *CYP72C1*. *CYP72C1* binds to the BL precursor to inactivate it and maintain BR homeostasis, and *CYP734A1* inactivates CS and BL by C-26 hydroxylation [13,14]. The results of a study using a cell-free extract from cultured *Phaseolus vulgaris* cells suggest that P450 enzymes are involved in the conversion of CS to BL [15]. Using reporter gene fusion, *Dwarf* has been shown to be expressed both in vegetative tissues and in fruits. A null allele, extreme dwarf (dx), shows severely reduced stem length, altered leaf morphology, and no detectable CS in vegetative tissues [16]. Previous reports indicate that auxin signaling via *ARF7* directly modulates the expression of *BAS1* by competition with *BZR1*, thereby increasing the level of CS and promoting growth and development in *Arabidopsis thaliana* [17]. Recent reports have demonstrated that higher levels of CS contribute to plant growth and development and increase plant biomass [7,18]. More recently, Song and Duan [19] regulated the activity of antioxidant enzymes by overexpression of *SoCYP85A1*, which increased CS accumulation and enhanced black shank tolerance in tobacco.

One of the most critical steps in the BR pathway is the conversion of 6-deoxoCS to CS [20]. P450 is a class of enzymes involved in oxidative reactions occurring during BR biosynthesis [21]. The *CYP85A1* gene encodes a cytochrome P450 enzyme containing monooxygenase *CYP85A1*, which is a necessary catalyst for several essential reactions required for CS production [22]. The synthesis of CS and BL requires the enzyme *CYP85A2*, which also contributes to the conversion of 6-deoxyCS into CS and finally into BL. Several lines of evidence show that altering CS content affects plant phenotypes. For example, extreme dwarfism in cucumber (*Cucumis sativus* L.) was found to be due primarily to the inhibition of internode elongation caused by mutation in *scp-1*/*CsCYP85A1* [23]. In another recent study, increasing the abundance of *SoCYP85A1* transcript and endogenous CS in transgenic tobacco resulted in longer main roots and more lateral roots in comparison with control plants, which enhanced drought resistance [24]. Simultaneously, Li et al. [25] found that overexpression of the *CYP85A1* gene in tomatoes increased the CS content in plants while enhancing the quantum yield of photosystem II and the CO_2_ assimilation rate. Therefore, increasing the expression of *CYP85A1* will likely increase endogenous CS content, which should enhance plant resistance to stressors, promote growth and increase biomass.

Among trees, poplar (genus *Populus*) has the largest planting area and widest distribution globally due to its strong adaptability. Poplar is an economically important group of species that play an important role in ecological protection. *Populus tomentosa* generally grows upright and has wood that is compact and delicate with very high fiber content, making it particularly useful for plate making, paper making, and other industrial applications. Previous studies have shown that modifying BR biosynthesis genes such as *DWF4* and *CYP85* enhances growth, biomass accumulation, and timber quality [26,27,28]. *OsCYP85A1*/*OsBRD1* is a key gene for CS synthesis in rice, which plays an important role in its rapid growth. *OsCYP85A1*/*OsBRD1* is a bromodomain-containing gene. Brd-containing proteins (alone or as multi-protein complexes) are involved in the regulation of gene expression by different mechanisms viz. chromatin remodeling, histone modifications, and transcriptional machinery regulation [29]. *Populus tomentosa* is an important industrial tree species; however, it took a relatively long time to develop into industrial wood [17]. In this study, the key gene *OsCYP85A1* of rice castasterone (CS) biosynthesis was genetically transformed into *P. tomentosa* to obtain transgenic poplars with the developmental characteristics of gramineous plants. Compared with the WT poplar, transgenic poplars can simultaneously accumulate a large amount of lignin. Their rate of growth is substantially improved so that the trees quickly become woody. Thus, they meet the needs for daily life and industrial production in the future, which have the potential to improve the production of materials and utilize land resources.

## 2. Results

### 2.1. Bioinformatic Analysis of CYP85A Gene and Expression Patterns in Poplar

**Figure 1:**
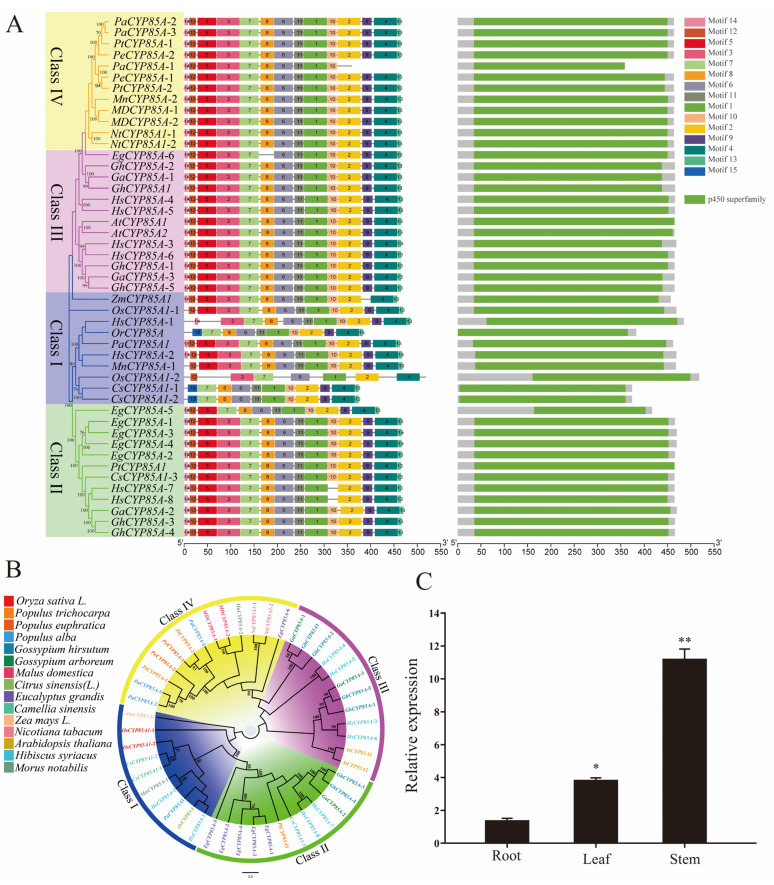
Sequence analysis of CYP85As proteins and expression patterns of *OsCYP85A1* in different plant parts. (**A**) *OsCYP85A* protein has the typical functional domains of a conserved cytochrome P450 monooxygenase. (**B**) Phylogenetic relationships of *OsCYP85A* with CYP85As from *P. euphratica* (Pe), *P. trichocarpa* (Pt), *A. thaliana* (At), *G. hirsutum* (Gh), *Zea mays* (Zm), *Oryza sativa* (Os), *Malus domestica* (MD), *Camellia sinensis* (Cs), *Gossypium arboretum* (Ga), *Nicotiana tabacum* (Nt), *Citrus sinensis* (CS), *Eucalyptus grandis* (Eg), *Hibiscus syriacus* (Hs), and *Morus notabilis* (Mn). The scale bar represents 0.05 substitutions per site. (**C**) Expression of *OsCYP85A1* in different organs of *P. tomentosa*. Error bars represent ± SD from three biological repeats. * Significant at *p* < 0.05 level and ** significant at *p* < 0.01 level according to Student’s *t*-test.

Two CYP85 members, *CYP85A1* and *CYP85A1*-like, were identified in *Oryza sativa* L., and their homologues in *Populus tomentosa*, *Populus euphratica*, *Populus trichocarpa*, *Gossypium hirsutum*, *Malus domestic*, *Camellia sinensis*, *Gossypium arboreum*, *Nicotiana tabacum*, *Zea mays*, *Citrus sinensis*, *Eucalyptus grandis*, *Hibiscus syriacus*, *Morus notabilis,* and *Arabidopsis thaliana* were searched in the NCBI database (Figure 1B). CYP85A in *O. sativa* and *P. tomentosa* is conserved with regard to protein length, molecular weight, isoelectric point (pI), and sequence similarity (Table S1). Interestingly, *OsCYP85A1* and *PtCYP85A*-2 have 67.25% similarity, with the same size and similar pI. *OsCYP85A1* was selected for further analysis and named *OsCYP85A1*-1. *OsCYP85A1*-1 had 65.86% similarity and 83.93% similarity to *PtCYP85A* and *ZmCYP85A*, respectively (the sequence alignment of *P. tomentosa* and *Z. mays* is shown in Figure S1), and the presence of several functional domains indicated that these proteins had similar functions (Figure S5). The *OsCYP85A* protein possesses the typical functional domains of a conserved cytochrome P450 monooxygenase: purine-rich domain, dioxygen-binding domain, steroid-binding domain, and heme-binding domain (Figure 1A). The results also showed that several cis-acting elements, including light and hormone response and circadian rhythm elements, were present in the *OsCYP85A* promoter (Figure S5). To further clarify the role of overexpression of *OsCYP85A1* on *Populus tomentosa*, we isolated total RNA from different organs from 3-month-old *P. tomentosa* and analyzed the transcript abundance of *OsCYP85A1* by quantitative real-time PCR (qRT-PCR) (Table 1). The expression level of *OsCYP85A1* in the stems and leaves was higher than that measured in the roots, which indicated that it played an important role in the growth and development of the aboveground parts of transgenic poplars (Figure 1C). Figure 1C shows the expression analysis and comparison with wild-type controls.

### 2.2. Overexpression of OsCYP85A1 Improves Plant Growth Development

#### 2.2.1. Generation of *OsCYP85A1* Transgenic *P. tomentosa* Lines

**Figure 2:**
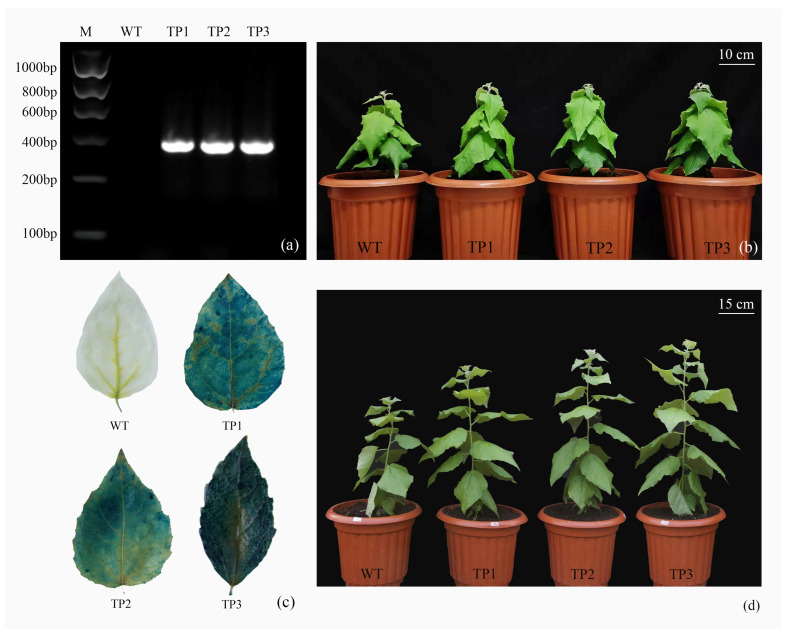
Molecular analyses of *OsCYP85A1* transgenic trees. (**a**) PCR analysis of wild-type and different independently generated transgenic lines. (**b**) Phenotypes of wild-type and three independent *OsCYP85A1* transgenic lines grown in a glass greenhouse for 7 weeks. (**c**) GUS staining of *OsCYP85A1* transgenic plants. (**d**) The 70 d transgenic plants.

To assess the potential value of *OsCYP85A1* in improving biomass production in woody plants, a pCAMBIA130::*OsCYP85A1*::GUS recombinant plasmid was constructed and transformed into *P. tomentosa.* The length of *OsCYP85A1* is 1436 bp. Six transgenic lines were obtained, and three lines with a high transcription level of *OsCYP85A1* were selected for further experiments: TP1, TP2, and TP3 (Figure 2b). PCR analysis confirmed that *OsCYP85A1* was integrated into the poplar genome (Figure 2a). Further analysis by GUS staining (Figure 2c) confirmed the overexpression of *OsCYP85A1* in all selected trans-genic lines. Simultaneously, the overexpression of *OsCYP85A1* in transgenic lines was ver-ified by qRT-PCR. On the 70th day of growth, the height of the transgenic lines was significantly higher than that of WT (Figure 2d).

#### 2.2.2. Growth Rate

**Figure 3:**
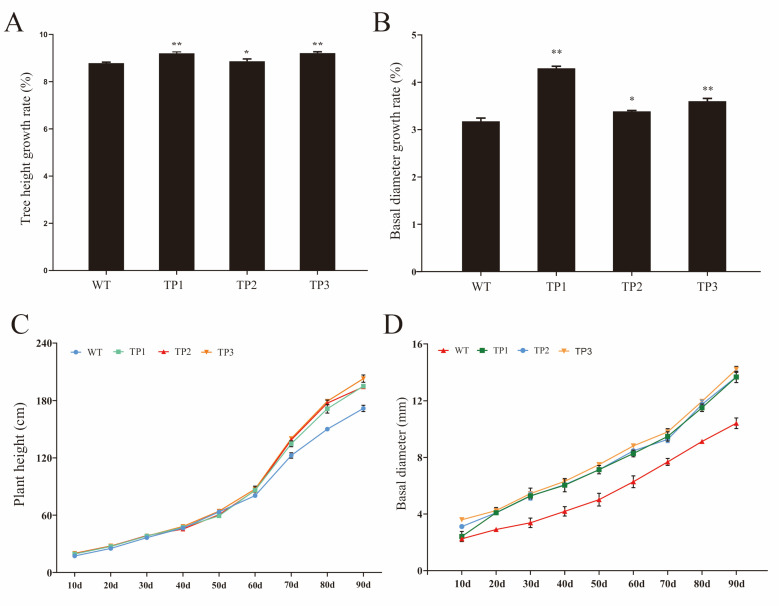
Overexpression of *OsCYP85A1* in poplar increases plant height and stem diameter. (**A**) Tree height growth rate. (**B**) Basal diameter growth rate. (**C**) Plant height. (**D**) Basal diameter. Error bars represent the SDs from three biological replicates. * and ** indicate significant differences in comparison to WT at *p* < 0.05 and *p* < 0.01, respectively (Student’s *t*-test).

The plant height and basal stem size affect plant architecture, lodging resistance, yield, and even the optimal plant harvesting strategy [30]. Changes in the height and basal stem growth of transgenic and wild-type *P. tomentosa* plants were recorded between 27 November 2021 and 27 March 2022. No significant differences in plant height were ob-served among the TP1, TP2, TP3, and WT lines for the first 70 days of observation. After 70 days, the TP1, TP2, and TP3 plants were significantly taller than the WT plants (Figure 3C). However, the basal stems of the TP1, TP2, and TP3 plants were significantly thicker than those of the WT plants after day 20 (Figure 3D). The height growth rate (HGR) and basal diameter growth rate (DGR) of the TP1 and TP3 transgenic lines were significantly greater than those of the WT plants (HGR: 1.05 times WT for TP1 and 1.06 times for TP3; DGR: 1.39 times WT for TP1 and 1.17 times WT for TP3). In addition, the HGR and DGR of TP2 were significantly greater than those of the WT plants (HGR: 1.02 times WT; DGR: 1.15 times WT) (Figure 3A,B).

#### 2.2.3. Effects of *OsCYP85A1* on Chlorophyll Content

**Figure 4:**
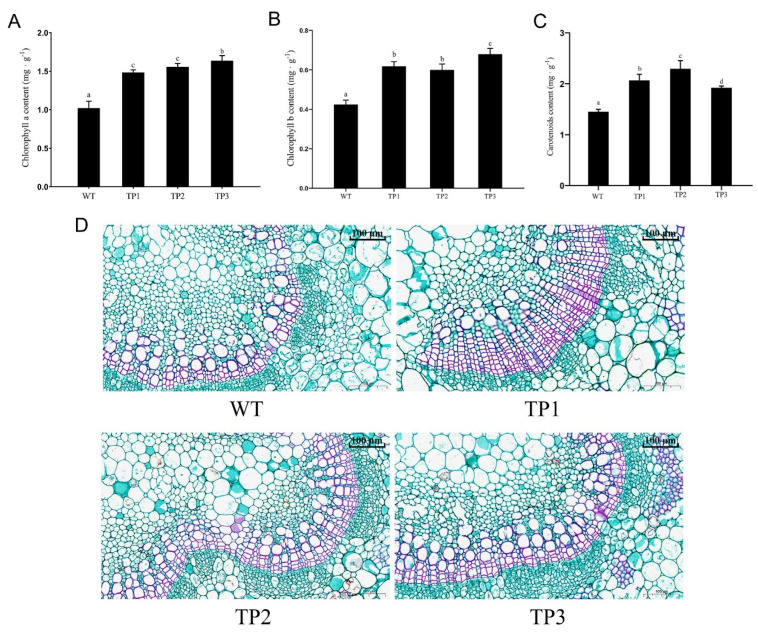
Effects of *OsCYP85A1* on the photosynthetic pigment content and petiole morphological characteristics of transgenic plants. (**A**) Chlorophyll a. (**B**) Chlorophyll b. (**C**) Carotenoid. (**D**) Morphological characteristics of transverse section of petiole per unit area of transgenic plants. Error bars represent mean ± SD of three biological replicates. Different letters indicate a significant difference using the Tukey–Kramer test: *p* < 0.05.

Li et al. [25] generated transgenic tomato plants by overexpressing *Dwarf*, a BR biosynthetic gene that encodes *CYP85A1*, and compared the photosynthetic capacity of the BR biosynthetic mutant dim and WT. Overexpression of *dwarf* increased the net photosynthetic rate (*PN*) and photosynthetic capacity by inducing a reduced redox status that maintained the activation states of Calvin cycle enzymes, thereby promoting plant growth and development. The leaves of TP1, TP2, TP3, and WT plants were collected at the same growth stage and from the same part of each plant for analysis of the content of photosynthetic pigments. The analysis revealed a significant difference between the photosynthetic pigment content of TP1, TP2, and TP3 in comparison with that of WT plants (Figure 4A–C). The results of this study were consistent with those of Li et al. [25]. At the same time, the petioles of each group, again collected at the same stage and from the same position, were subjected to anatomical analysis (Figure 4D). The transverse sections of petioles in each group were morphometrically analyzed by Fiji/ImageJ software (Fiji- ImageJ version of Java 6, NIH, Bethesda, MD, USA) (Figure S2A). The results indicated that the TP2 and TP3 petioles had significantly more vessels and sieve tubes in comparison with WT petioles; however, the number of vessels in TP1 was slightly lower than that in WT, but the total number of vessels and sieve tubes in TP1 was also significantly higher than that in WT (Figure S2B,C). Similarly, the number and area of vascular bundles in the whole petiole cross-section of transgenic lines were greater than those of the wild type (Figure S4), which likely facilitated the transportation of water and nutrients in the transgenic plants, promoting increased biomass. Red is the result of safranine staining and decolorization, and green is the result of fixation green staining and decolorization.

### 2.3. Overexpression of OsCYP85A1 Regulates Root Development

**Figure 5:**
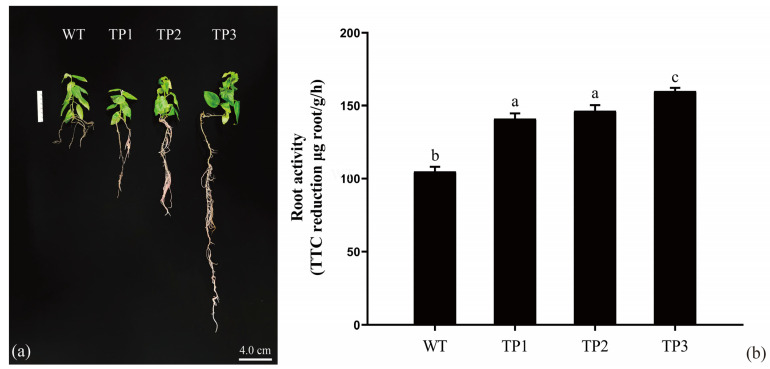
The effect of *OsCYP85A1* overexpression on the root development of transgenic plants. (**a**) Phenotype of 5-week-old seedlings under normal conditions. (**b**) The metabolic activity of transgenic plant roots was estimated by tetrazolium chloride (TTC) reduction. Error bars represent mean ± SD of three biological replicates. Different letters indicate a significant difference using the Tukey–Kramer test: *p* < 0.05.

The root development of TP1, TP2, TP3, and WT plants was assessed at 5 weeks and 6 months of age. The roots were grown under the same culture conditions in a tissue culture room (relative humidity of 60–70%, 16 h/8 h light/dark cycle, and 25 °C). In comparison with the WT line, the main roots of the transgenic lines were longer and had a greater number of lateral roots after 5 weeks (Figure 5a). After 6 months, the entire plant was harvested, and all roots were washed. Root segments of the same position and length were selected, and root activity was determined by the TTC method (Figure 5b). A significant difference in root activity was observed between transgenic and WT roots. Higher root activity helps transgenic plants absorb inorganic salts and water from the soil, thus pro-moting the growth of their aboveground parts [24]. The growth and biomass of transgenic plants were significantly increased in this study, indicating that *OsCYP85A1* has great po-tential for molecular breeding of fast-growing tree species by regulating BR production in poplar and other woody plants.

### 2.4. Overexpression of OsCYP85A1 Enhanced Plant Disease Resistance

**Figure 6:**
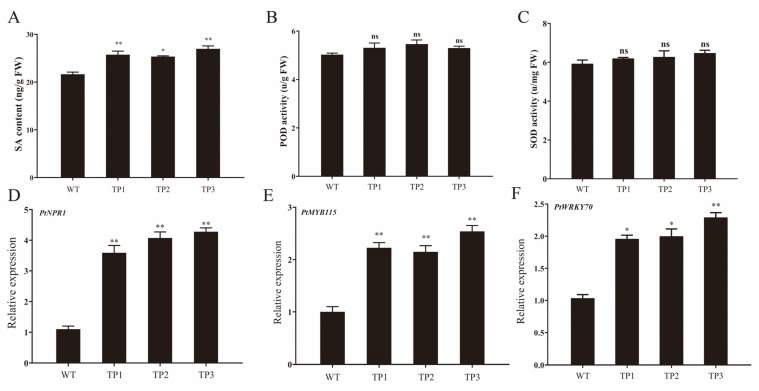
Overexpression of *OsCYP85A1* enhanced plant disease resistance. (**A**) Salicylic acid (SA); (**B**) Superoxide dismutase (SOD); (**C**) Peroxidase (POD). Expression of genes related to poplar disease resistance. The expression level of these genes in the stem of WT was set to 1. ns, nonsignificant; Error bars represent the SDs from three biological replicates. * and ** indicate significant differences in comparison to WT at *p* < 0.05 and *p* < 0.01, respectively (Student’s *t*-test).

According to previous reports, the contents of disease-resistant metabolites in the leaves of transgenic lines and WT after 90 d of growth were determined, and the expression of disease-resistant genes in plants was analyzed. The results showed that the salicylic acid content of transgenic lines was significantly higher than that of wild type, while the activities of superoxide dismutase (SOD) and peroxidase (POD) were not significantly different from those of the wild type (Figure 6D–F). Interestingly, the expression levels of resistance-related genes *PtNPR1*, *PtMYB115,* and *PtWRKY70* in transgenic lines were significantly higher than those in the wild type (Figure 6D–F). We speculate that this is related to the bromodomain-containing protein encoded by *OsCYP85A1*/*OsBRD1*. This is the same as the previous research results; the increase in BR content will enhance the disease resistance of plants [31].

### 2.5. OsCYP85A1 Promotes Xylem Differentiation in Transgenic Plants

**Figure 7:**
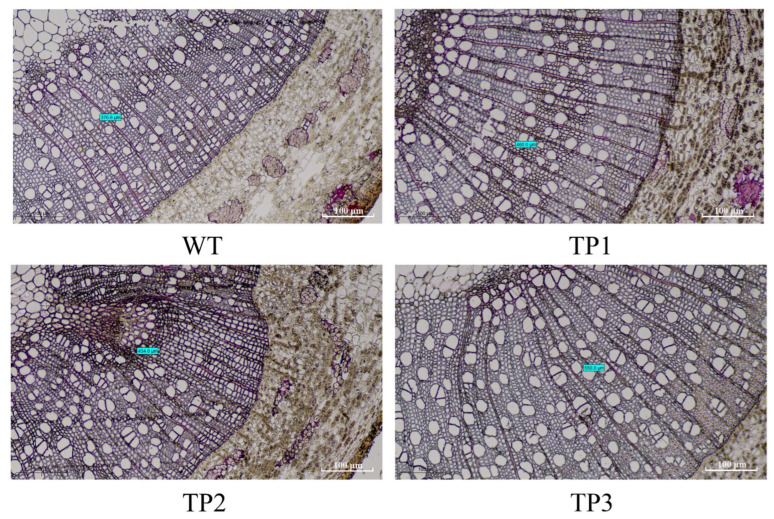
Cross-sections of stems showing increased xylem area in 90 d old wild-type and transgenic plants (lines 1, 2, and 3). Blue is marked as the thickness of xylem per unit area. WT. The thickness of xylem is 376.6 μm. TP1. The thickness of xylem is 450.0 μm. TP1. The thickness of xylem is 434.0 μm. TP1. The thickness of xylem is 550.0 μm.

**Figure 8:**
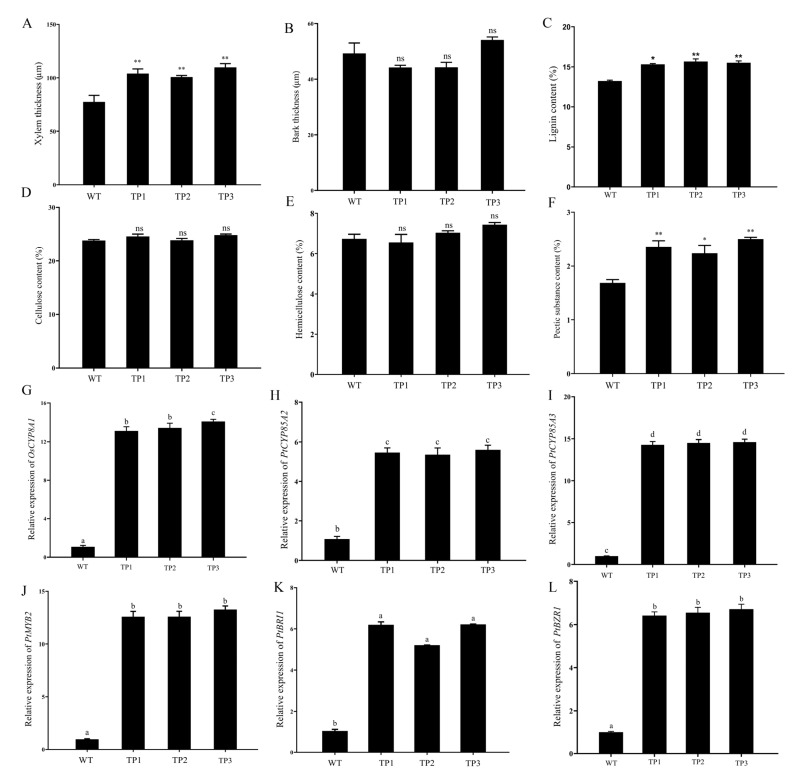
Effects of *OsCYP85A1* on xylem differentiation in transgenic poplar. (**A**,**B**) Measurement of xylem and bark. (**C**,**D**) Cellulose and lignin contents in the xylem tissues. (**E**,**F**) Hemicellulose content and pectic content. (**G**–**L**) Expression analysis of secondary cell wall synthesis-related MYB transcription factors and CS synthesis-related genes. The expression level of these genes in the stem of WT was set to 1. ns, nonsignificant; error bars represent the SDs from three biological replicates. * and ** indicate significant differences in comparison to WT at *p* < 0.05 and *p* < 0.01, respectively (Student’s *t*-test). Error bars represent mean ± SD of three biological replicates. Different letters indicate a significant difference using the Tukey–Kramer test: *p* < 0.05.

To study the effect of *OsCYP85A1* on the secondary growth process of woody plants, the stems of WT and transgenic plants were microscopically analyzed. As shown in Figure 7 xylem growth was improved in all tested *OsCYP85A1* transgenic lines. Transgenic plants produced more xylem (Figure S3), but there was no significant difference in the yield of bark between the WT and transgenic plants (Figure 8A–D). There was no significant difference in the content of hemicellulose between the transgenic lines and WT, but the content of pectin was significantly higher than that of the WT (Figure 8E,F). Further studies were carried out on *PtMYB2*, an MYB transcription factor regulating secondary cell wall synthesis in transgenic plants, as well as *PtCYP85A2*, *PtCYP85A3*, *PtBRI1*, and *PtBZR1*, which control CS and BL biosynthesis in *P. tomentosa* (Figure 8G–L). The results of these experiments indicated that *OsCYP85A1* participates in secondary cell division and promotes the anabolism of CS and BL during poplar wood formation, thus promoting the growth and wood formation of transgenic plants.

## 3. Discussion

Current studies have fully demonstrated that *CYP85A1* is necessary for the production of CS. Until now, functional studies of *CYP85A1* have been carried out in *Arabidopsis*, tomato, and rice, but few studies have assessed the role of this gene in *P. tomentosa* [24]. In this study, *CYP85A1* was observed to have a highly conserved cytochrome P450 domain, indicating that *CYP85A1* belongs to the P450 superfamily. Multiple sequence alignment showed that *OsCYP85A1* has high sequence consistency with *CYP85A1* genes from other plants at the amino acid level. Phylogenetic tree analysis revealed the relationship between *OsCYP85A1* and *CYP85A1* genes from other plants, showing that *OsCYP85A1* is closely related to *CYP85A1* from *Zea mays* (NM_001349820.1, 83.93%) and *P. tomentosa* (EEE85904, 65.86%). Numerous studies have shown that *CYP85A1* regulates biocatalysis, plant growth, and development [21,25]. However, no research has been published on the effects of *CYP85A1* from herbaceous plants on the growth and development of woody plants, which could provide new ideas for the creation of novel industrial tree species.

The qRT-PCR data showed that the *OsCYP85A1* transcript was more abundant in stems and leaves. These results were different from those of previous studies, which reported that CYP85A1 mRNA mainly accumulated in roots or buds [16,32]. The discrepancy between these results may be due to the use of different species and different sample collection times [24]. Alternatively, because the gene is expressed under the control of the constitutive promoter CaMV 35s, the expression in leaves and stems is higher than that in roots and buds [33]. High expression of *OsCYP85A1* in young leaves and stems suggests that it plays a key role in BR metabolism and the normal growth of trees. Under the control of the CaMV 35S promoter, transgenic poplars were generated by Agrobacterium-mediated transformation with *OsCYP85A1*. The agronomic traits of three selected transgenic lines (TP1, TP2, and TP3) were analyzed. In comparison with WT plants, the transgenic lines had longer main roots, more lateral roots, higher plant height, and thicker stems. These findings are consistent with the results of Li et al. [25]. *DWARF* is a key gene involved in BR synthesis in tomatoes. When DWF transcripts and endogenous BR levels are increased, germination, lateral root development, CO2 assimilation, and final plant growth are improved, as demonstrated by elongation and compactness of plant structure. Consistent with Kim et al., *CYP85A2* overexpression enhanced the growth of *Arabidopsis thaliana*; *CYP85A2* overexpression induced higher levels of BL, conferring transgenic plants with significant advantages in morphogenesis, leading to larger rosette leaves and longer petioles in comparison with those of WT plants [34]. Therefore, genes involved in BR biosynthesis can be manipulated to control plant growth, photosynthesis, structure, stress resistance, and other important agronomic traits, thus improving crop yield [35].

Previous reports on the involvement of endogenous BR in promoting wood formation and the abnormal xylem appearance of BR dwarf mutants suggest that BR biosynthesis occurs in vascular tissue [20,36]. Research by Hamasaki et al. [37] showed that light activates the expression of BR biosynthesis genes in the hook region via a phytochrome-signaling pathway and HY5, and BR biosynthesis was shown to be essential for hook opening and petiole development during photomorphogenesis. Genes involved in BR synthesis include *CYP90A1*, *CYP90C1*, and *CYP90D1*, which regulate C-23 hydroxylation shortcuts and camphor-independent pathways, and *BR6ox1*/*CYP85A1* and *BR6ox2*/*CYP85A2*, which control the biosynthesis of CS and BL [16,38]. In this study, *OsCYP85A1*, which controls the synthesis of CS in rice, was transformed into *P. tomentosa*. Histological analysis showed that the petioles of transgenic plants had significantly more vessels and sieve tubes in comparison with those of WT plants, which helped transgenic plants transport more organic matter to roots and more inorganic salts and water to leaves.

Physiological, genetic, and molecular studies have revealed the roles of plant hormones in xylogenesis and vascular tissue differentiation. The expression levels of some genes involved in cell wall synthesis were found to be reduced in BR synthetic mutants, such as XETs and MERI5 in *dwf1* [39]. In addition, brassinazole (Brz) was found to be capable of suppressing the development of the secondary xylem of cress plants [40]. Another recent study showed that overexpression of *PtCYP85A3*, a functional allele of *AtCYP85A2* and *SlCYP85A1*, increased the yield of CS and regulated secondary cell division and fiber elongation during poplar wood formation, thereby promoting growth and wood formation [26]. Overexpression of *PtBRI1*, a brassinosteroid-insensitive gene, promotes the accumulation of *PtBZR1* (BRASSINAZOLE RESISTANT1) in the nucleus and then activates *PtWNDs* (*WOOD-ASSOCIATED NAC DOMAIN* transcription factor) to regulate the expression of secondary cell wall biosynthesis genes involved in wood formation; this process plays a crucial role in regulating twig growth and wood formation [17]. In this study, we found that transgenic poplars produced more xylem than wild-type plants, but the increase in the bark was not significant. The expression levels of secondary cell wall-related transcription factor genes, genes controlling BR biosynthesis in poplar, and genes regulating xylem synthesis were significantly up-regulated in transgenic plants. These results indicate that *OsCYP85A1* can participate in both secondary cell division and fiber elongation during poplar wood formation. Overexpression of *OsCYP85A1* increased CS production, thereby promoting the growth and wood formation of transgenic plants.

Previous studies have shown that *CYP85A1* is mainly expressed in roots and hypocotyls, specifically at the root-hypocotyl junction during early development [20]. A common feature of the data obtained from the CYP85 promoter is that the highest expression level occurs early in development, after which expression is down-regulated over time [32]. In this study, it was found that the primary root length of the transgenic plants was 5.2 times that of the wild type, and the number of lateral roots was also significantly higher than that of the wild type. However, when detecting the expression of *OsCYP85A1* in the roots, stems, and leaves of transgenic plants after 90 days of growth, it was found that the expression of *OsCYP85A1* in roots was significantly lower than that in stems and leaves. These results are consistent with the above research. Overexpression of *SoCYP85A1* enhanced drought tolerance in transgenic tobacco with increased CS content, which promoted root development and led to the accumulation of a higher relative water content (RWC) and proline, as well as less malondialdehyde (MDA) and H_2_O_2_, improved the activities of antioxidant enzymes, and increased the transcript expression of ROS-related and stress-responsive genes under drought stress [24]. In summary, *CYP85A1* has a positive effect on plant root development [41]. In order to verify that *OsCYP85A1* plays an important role in the early stage of plant development, we measured root activity using the TTC method after 90 days of growth. The results showed that the root activity of the transgenic plants was significantly higher than that of the wild type, which likely helped the transgenic plants absorb more nutrients and promote growth and development.

## 4. Materials and Methods

### 4.1. Bioinformatics Analysis

The protein sequences of *Oryza sativa* L. CYP85A family members were used as queries to search against the reference protein sequence databases of *Populus tomentosa*, *Populus euphratica*, *Populus trichocarpa*, *Gossypium hirsutum*, *Malus pumila*, *Camellia sinensis*, *Gossypium arboretum*, *Nicotiana tabacum*, *Zea mays*, *Citrus sinensis*, *Eucalyptus grandis*, *Hibiscus syriacus*, *Morus notabilis*, and *Arabidopsis thaliana* (available at the National Center for Biotechnology Information) using BLASTP (http://www.ncbi.nlm.nih.gov/BLAST/, accessed on 31 January 2022). The results were combined and splice variants were removed. Sequence similarity was calculated by Sequencher v4.7. The molecular weight and theoretical isoelectric point (pI) of the proteins were predicted with ProtParam (http://web.expasy.org/protparam/, accessed on 8 June 2022). A phylogenetic tree was built with MEGA 6.0 software according to the neighbor-joining statistical method with 1000 bootstrap replicates. Multiple sequence alignments were generated with DNA-MAN 7.0. Conserved domains and motifs were analyzed by CDD in the NCBI (http://www.ncbi.nlm.nih.gov/Structure/cdd/docs/cdd_search.html, accessed on 8 June 2022). The promoter sequence of the *OsCYP85A* gene was analyzed by the online software Plant CARE (http://bioinformatics.psb.ugent.be/webtools/plantcare/html/, accessed on 27 June 2022) to predict cis-acting elements. The above experiments were carried out in the Tea College of Guizhou University (26°44′ N, 106°65′ E).

### 4.2. Materials and Transformation

Plant materials: in this study, *P. tomentosa* hybrid poplar (*P. tomentosa* Carr. (♀) × *P. simonii* Carr. (♂)) seedlings were used as the experimental material. Strains and vector plasmids: *Escherichia coli* DH5α, *Agrobacterium tumefaciens* LB4404; CaMV 35S promoter cloning vectors pCAMBIA130::*OsCYP85A1*::GUS. Carrier construction method: the *OsCYP85A1* gene sequence was obtained by PCR amplification using rice cDNA as a template and high-fidelity polymerase PrimeSTAR^®^ Max (Junotec Biotechnology Co., Ltd., Wuhan, China). The plasmid vector pCAMBIA130 was digested with *Xba*I and *BamH*I, and the synthetic target fragment *OsCYP85A1* was digested with *Xba*I and *Kpn*I. pCAMBIA130 and *OsCYP85A1* were ligated by T4 DNA ligase (Covent Biotechnology Co., Ltd., Changsha, China). The pCAMBIA130::*OsCYP85A1*::GUS vector driven by the constitutive promoter CaMV 35S was constructed. The β-glucuronidase (GUS) fusion gene was used as a screening marker and reporter gene. The above materials were stored in the College of Tea Sciences at Guizhou University (26°44′ N, 106°65′ E), Guiyang, China. *P. tomentosa* transformation was implemented by the Agrobacterium-infected leaf disc method. Infected Agrobacterium LB4404-leaf discs were first cultured in MS culture medium (MS) with 1.0 mg·L^−1^ naphthylacetic acid (NAA) and 1.0 mg·L^−1^ zeatin (ZT) in the dark for 2 days, after which they were placed into MS with 1.0 mg·L^−1^ NAA, 1.0 mg ·L^−1^ ZT, 50 mg ·L^−1^ kanamycin, and 200 mg·L^−1^ timentin (TIM) and kept in the dark for 2–3 days until callus formation. Subsequently, the calluses were switched to MS with 1.5 mg·L^−1^ ZT, 25 mg·L^−1^ kanamycin, and 200 mg·L^−1^ TIM and cultured under a 16/8 h light/dark cycle to induce shoot regeneration. Finally, the shoots (about 1 cm in length) were cut down and cultured in 1/2 MS with 0.2 mg·L^−1^ NAA, 50 mg·L^−1^ kanamycin, and 200 mg·L^−1^ TIM. Regenerated roots formed after about 1 week.

### 4.3. Quantitative PCR (qPCR) and Gus Histochemical Staining

The genomic DNA of rooted plantlets was extracted by the CTAB method [42] to confirm whether the recombinant plasmid had been successfully inserted into the plants using PCR (forward primer *OsCYP5A1*-F and reverse primer *OsCYP5A1*-R; see Table 1). The first leaves of 1-month-old plantlets were subjected to Gus histochemical staining and serial decolorization in 10%, 25%, 50%, 75%, and 95% ethanol at 37 °C until the leaves turned white, after which they were observed and imaged under a stereoscopic microscope.

### 4.4. Determination of Growth Indices

#### 4.4.1. Growth Measurement

Healthy poplars without pests and diseases that had grown for 90 d were selected as experimental materials [26]. Sapling height was measured using a tape rule from the base of the stem to the terminal bud. Stem basal diameter was measured by a Vernier caliper at the base of stem. Sapling height and stem basal diameter measurements were taken on 27 November 2021 and 27 March 2022, respectively. The relative growth rate of height (HGR) and basal diameter (DGR) were calculated using Equations (1) and (2) [43], where *H*_1_ is initial sapling height (cm), *H*_2_ is final sapling height (cm), *D*_1_ is initial basal diameter (mm), and *D*_2_ is final basal diameter (mm).

(1) $$HGR=\left(H_{2}-H_{1}\right){/H}_{1}$$

(2) $$DGR=\left(D_{2}-D_{1}\right){/D}_{1}$$

#### 4.4.2. Determination of Photosynthetic Pigments

Wild-type (WT) and transgenic plants (TP1, TP2, and TP3) were selected as experimental materials, and the third functional leaf of each plant was collected. Fresh leaves (0.1 g) without the midrib were weighed, ground into a powder, and soaked in 10 mL of 95% alcohol to extract the pigment. The specific process followed the method of Ren et al. [44]. The absorbance values (*A*_665_, *A*_649_, and *A*_470_) of each extract at 665 nm, 649 nm, and 470 nm were measured using a UH5300 spectrophotometer (Hitachi Co., Ltd., Tokyo, Japan). Each treatment was repeated three times. The concentrations and contents of chlorophyll a (*Ca*), chlorophyll b (*Cb*), and carotenoids (*Car*) were calculated using Equations (3)–(5).

(3) $$Ca=13.95A_{665}-6.88A_{649}\;$$

(4) $$Cb=24.96A_{649}-7.32A_{665}$$

(5) $$Car=\frac{{1000\;A}_{470}-2.05\;Ca\;-114.8\;Cb}{245}$$

#### 4.4.3. Lignin Content Determination

Quantification of lignin was performed via the acetyl bromide method [45]. Fresh samples of *P. tomentosa* stem segments (1 g) were placed into a screwcap centrifuge tube containing 2 mL of 25% acetyl bromide (*v*/*v* in glacial acetic acid) and incubated at 70 °C for 30 min. After complete digestion, the sample was quickly cooled in an ice bath and mixed with 0.9 mL of 2 M NaOH, 0.1 mL of 5 M hydroxylamine-HCl, and a volume of glacial acetic acid sufficient for complete solubilization of the lignin extract. After centrifugation (4500 rpm, 5 min), the absorbance of the supernatant was measured at 280 nm. The absorbance value was used to calculate the relative content of lignin per gram of fresh sample.

#### 4.4.4. Determination of Cellulose, Hemicellulose, and Pectin Content

The method of Peng et al. [46] was used to determine the contents of cellulose, hemicellulose, and pectin in the stem of *P. tomentosa.* Ammonium oxalate was used to extract pectin. The obtained crude cell wall precipitate was added to 5 mL of 0.5% ammonium oxalate, heated in boiling water for 1 h, and centrifuged, after which the supernatant was collected. The precipitate was washed with 5 mL 0.5% ammonium oxalate once, after which it was washed twice with distilled water. All supernatants were collected and diluted to a constant volume, and pentose, hexose, and galacturonic acid were determined by colorimetry. Galacturonic acid reacted with 3-phenyl phenol to form a colored dye, and the absorbance was measured at 530 nm. Pectin content was expressed as galacturonic acid content (%) [47]. Alkali-soluble hemicellulose was extracted by 4 M KOH. First, 5 mL of 4 M KOH was added to the precipitate after the extraction of pectin by ammonium oxalate, and the sample was oscillated at 90× *g* for 1 h, shaken at 150× *g* for 1 h at 25 °C, and centrifuged at 4000× *g* for 5 min. The precipitate was washed once with 5 mL of 4M KOH and twice with distilled water. All supernatants were collected and diluted to a fixed volume of 10 mL. Pentose and hexose were determined by colorimetry. A total of 1 g of residue was put into a 10 mL measuring flask.. Sulfuric acid extraction was used to obtain total cellulose and alkali insoluble hemicellulose. First, 3.0 mL of 67% (*v*/*v*) sulfuric acid was added to the above residue and mixed. The sample was shaken at 150× *g* for 1 h at 25 °C, and distilled water was added to a volume of 10 mL. The sample was cooled to room temperature, centrifuged at 4000× *g* for 5 min, and the supernatant was collected. Finally, pentose and hexose were determined by Sulfuric Acid–UV method [48]. The hexose results reported in this step indicate total cellulose, whereas pentose consists of alkali-insoluble hemicellulose, and total hemicellulose is the sum of alkali-soluble hemicellulose and alkali-insoluble hemicellulose. UV spectrophotometer was used to measure the UV absorption at 315 nm. Tamarind xylan was used as a standard for the preparation of calibration curves, and the results were expressed as hemicellulose content in the sample [49]. The UV absorption of hexose at 620 nm was also measured by UV spectrophotometer. Glucose was used as a standard for the preparation of calibration curves, and the results were expressed as the cellulose content in the sample [46].

### 4.5. Triphenyltetrazolium Chloride (TTC) Mothed Root Activity Determination and Disease Resistance Analysis

Root activity was analyzed by the triphenyl tetrazolium chloride (TTC) method. TTC is a chemical that is reduced by dehydrogenases when added to a tissue. The dehydrogenase activity is regarded as an index of root activity. The specific process followed the method of Zhang et al. [50]. First, 0.5 g fresh *Populus tomentosa* root was immersed in 10 mL of a homogenous mixed solution of 0.4% TTC and phosphate buffer and kept in the dark at 37 °C for 2 h. Subsequently, 2 mL of 1 mol/L H_2_SO_4_ was added to stop the reaction with the root. The root was dried with filter paper and then extracted with ethyl acetate. The red extractant was transferred into a volumetric flask to reach 10 mL by adding ethyl acetate. The absorbance of the extract at 485 nm was recorded. Root activity was expressed as the TTC reduction intensity. Root activity = amount of TTC reduction (µg)/fresh root weight (g) × time (h). In order to avoid the influence of changes in root microbiome activity on experimental data, in this study, the collected root samples were soaked in 75% ethanol for 5 min, sterile water was used to clear 3~4 times, and the samples were dried before TTC experiment [51].

In accordance with previous studies, physiological parameters such as superoxide dismutase (SOD) and peroxidase (POD) of transgenic poplar and wild-type poplar were measured [52,53]. The total endogenous salicylic acid (SA) in poplar leaves was extracted according to the method described in previous reports [54,55]. The quantification of the total SA content in plant samples was performed with a plant SA ELISA test kit (DREP0874c, Kamai Shu, Shanghai, China), as described by the manufacturer. Each sample had three biological copies. The expression of disease-resistance-related genes *PtNPR1*, *PtMYB115*, and *PtWRKY70* was also analyzed [56].

### 4.6. Histological Analysis of OsCYP85A1 Transgenic Populus tomentosa and the Wild Type

In order to explore the stem tissue structure of transgenic poplar overexpressing *OsCYP85A1* and wild-type plants, fresh petiole segments of each sample were collected and immediately fixed in a formalin–glacial acetic acid–alcohol (FAA) solution containing 3.8% formalin, glacial acetic acid, and 70% alcohol (*v*:*v*:*v* = 1:1:9) for 24 h. During fixing, the air was extracted through a pump. The paraffin sections were prepared according to the method of Miao et al. [30]. The samples were dehydrated with a series of ethanol solutions and stained with 1% safranine. After they were embedded in paraffin, all cross-sections (20 μm) were cut and stained with 0.1% fast green. The specimens were observed and scanned using Pannoramic DESK (3DHISTECH, Budapest, Hungary). According to the phloroglucinol staining method of Rao et al. [57], the stems of the seventh section were stained, and the sections were decolorized, observed, and photographed under a NIKON Eclipse Ci (Nikon, Tokyo, Japan).

### 4.7. Analysis of Expression by qRT-PCR

*OsCYP85A1* (AB084385.1) encodes the protein responsible for BR C-6 hydroxylation in rice. To understand the possible functions of CYP85A family proteins in wooden plants, we performed a BLAST search in the Joint Genome Initiative poplar database (JGI, *Populus trichocarpa* genome portal v1.1; http://genome.jgi-psf.org/Poptr1_1/Poptr1_1.home.html, accessed on 31 January 2022) and identified two homologues in the Populus genome database in the CDS of *OsCYP85A1*, designated *PtCYP85A2* (EEF10243) and *PtCYP85A3* (EEF02250.2). Total RNA was extracted using a TRIzol-A+ kit (TianGen Biochemical Technology Co., Ltd., Beijing, China), cDNA was synthesized by reverse-transcription using a PrimeScript^TM^ II first strand cDNA Synthesis Kit (Solarbio Technology Co., Ltd., Beijing, China), and the cDNA was diluted by 10-fold for real-time PCR detection. To determine the expression levels of the *OsCYP85A1*, *PtCYP85A2*, *PtCYP85A3*, *PtBRI1* (XM_024597445.1), *PtBZR1* (XM_006375009.2), and *PtMYB2* (XM_002299875) genes, Primer Premier 5.0 was used to design specific primers for *OsCYP85A1*, *PtCYP85A2*, *PtCYP85A3*, *PtBZR1*, *PtBRI1*, and *PtMYB2*. The actin gene from *Populus trichocarpa* was used as an internal reference (Table 1). A real-time quantitative reverse transcription PCR (qRT-PCR) assay was performed on a Bio-Rad CFX Connect^TM^ real-time quantitative PCR instrument (Bio-Rad, Hercules, CA, USA). qRT-PCR was performed with a general-purpose, high-sensitivity, dye-based quantitative PCR detection kit from Nanjing Novozan Biotechnology Co., Ltd. (Nanjing, China). The qRT-PCR reaction system was utilized according to the manufacturer’s instructions. The conditions and system of qRT-PCR were as follows: 1 cycle of denaturation (94 °C, 5 min), followed by 40 cycles of amplification (94 °C, 30 s; 50 °C, 45 s), and signal acquisition (72 °C, 113 s). The reaction mixture included 50 µL of the qRT-PCR selection system, which consisted of 20 µL of nuclease-free water, 25 µL of Pfu PCR Mix (Biorun Biotechnology Co., Ltd., Wuhan, China), 1 µL of template, 2 µL of primer (+) (100 μM), and 2 µL of primer (−) (100 μM). The relative levels of expression of the genes were analyzed using the 2^−ΔCt^ method.

### 4.8. Data Analysis

The data were subjected to an analysis of variance (ANOVA) using SPSS 18.0 (IBM, Armonk, NY, USA), and the error bars represent the ±standard deviation of data from three independent experiments. The data were normalized, and all samples were normally distributed with homogeneity of variance. Different letters indicate significant differences at *p* < 0.05 and extremely significant differences at *p* < 0.01. The qRT-PCR data were analyzed using SPSS for significant difference analysis (*p* < 0.05). The data were analyzed using Microsoft Excel 2007 (Redmond, WA, USA), and a sequence analysis was performed using DNAMAN 5.0 and a public website (https://www.ncbi.nlm.nih.gov/, accessed on 8 June 2022) database.

## 5. Conclusions

This study showed that *OsCYP85A1* significantly increased the growth and biomass yield of transgenic plants. First, it affected the vascular tissue in the petiole. The numbers of vessels and sieve tubes in the petioles of transgenic plants were significantly higher than those of the wild type. The content of photosynthetic pigments in the leaves of the transgenic plants at the same position was higher than that of the wild type, which likely helped the transgenic plants to produce more organic matter for their own use. Second, transgenic poplar produced more xylem than wild-type plants, but the amount of bark in each line was similar. *OsCYP85A1* participated in secondary cell division during poplar wood formation. Finally, the effect of *OsCYP85A1* on the root system of transgenic plants showed a promoting effect in the early stage of transgenic poplar development. The main root of transgenic plants was significantly longer than that of WT plants. At the same time, the root activity of transgenic plants after 90 days of growth was also significantly higher than that of WT plants, which was conducive to the absorption of more water and inorganic salts by transgenic *P. tomentosa*, leading to enhanced growth and development. In summary, *OsCYP85A1* has great potential for the molecular breeding of fast-growing tree species due to its capability to regulate BR production in poplar and other woody plants. This study will provide potential candidate genes for economic tree species with long growth cycles and shorten the time to produce finished products for wood or other uses, which meet the needs for daily life and industrial production in the future.

## Figures and Tables

**Table 1:** List of primers used in this study.

Usage	Primer Name	Primer Sequence
PCR	*OsCYP85A1*	F: AACCTTCCTGGAACCAACTAC R: CGAAGATAACAGCTCGAGTGAA
qRT-PCR	*OsCYP85A1*	F: TGGAGGAGGTAGTCGAATGT R: CCTTCTTCCTCCCATCTGTATTG
qRT-PCR	*PtBRI1*	F: GATGTCAGAGGTGGTCAGAATG R: GGGTGGTGAGTGTGGTTAAA
qRT-PCR	*PtBZR1*	F: GGTTAAGGGCTCAAGGGAATTA R: CTGTGTCCCTTGCGATAAGTAG
qRT-PCR	*PtMYB2*	F: TTGGAGTGATGTAGCAAGGAA R: GATGAAGATGACAGTGACGGAT
qRT-PCR	*PtCYP85A2*	F: GAGAGCAAGGTATGGGAGTATTT R: GCCCTTTCCCTCGTTCATTA
qRT-PCR	*PtCYP85A3*	F: CTATCCAGAGCCTTCAACCTTC R: CCAGTTCCTTTCCAGGACATAG
qRT-PCR	*PtNPR1*	F: GTTGACCTAAATGAGACACC R: TAATCTCAGCCTTGTCCTTG
qRT-PCR	*PtWRKY70*	F: AATCCAAGGAGCTACTAC R: GTTACCATTGTTGTTGTGG
qRT-PCR	*PtMYB115*	F: GCCATTGGAGGTCTTTGCC R: GGTTACCGAGGAGGGAGTGC
qRT-PCR	Actin	F: GCATCCACGAGACTACATACAA R: TCAGCAATACCAGGGAACATAG

## Data Availability

Not applicable.

## Supplementary Materials

The following supporting information can be downloaded at: https://www.mdpi.com/article/10.3390/ijms24076480/s1.
