# Supplementary material for: The Overexpression of Oryza sativa L. CYP85A1 Promotes Growth and Biomass Production in Transgenic Trees
Source: Int J Mol Sci. 2023 Mar 30;24(7):6480. doi: 10.3390/ijms24076480 (PMC10095185; doi:10.3390/ijms24076480)
Supplement: Supplementary file 1 [file ijms-24-06480-s001.zip › supplementary.pdf]

Table S1. Information of *CYP85A* genes.

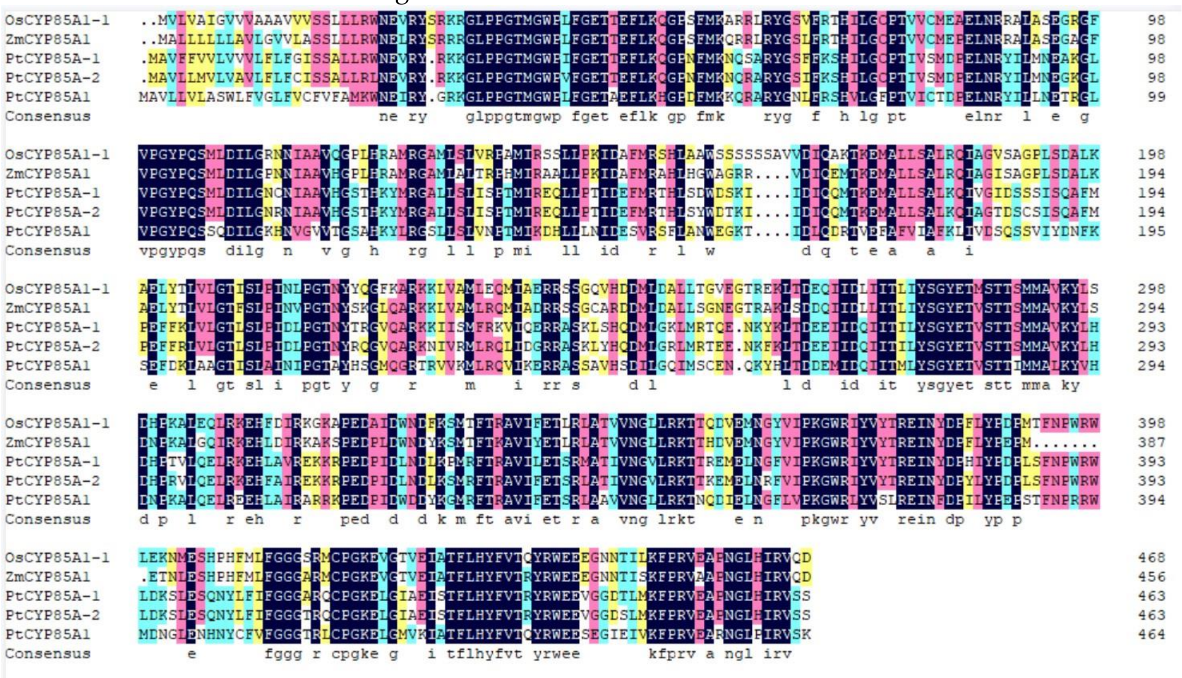

Figure S1. Sequence alignment of *Populus tomentosa* and *Z. mays*.

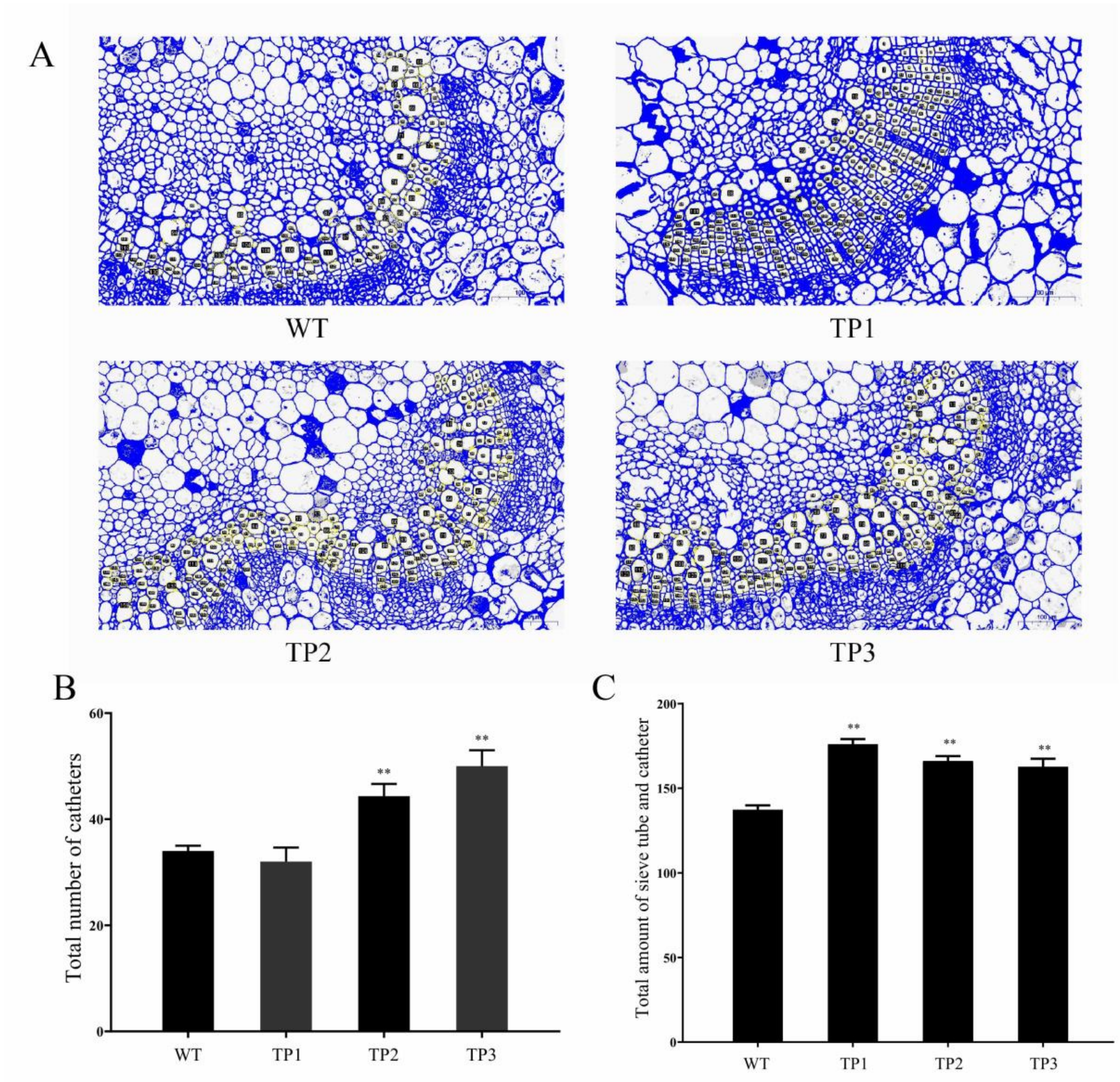

Figure S2. Morphometric analyses with the quantification of petiole cross sections of transgenic lines. **P < 0.01

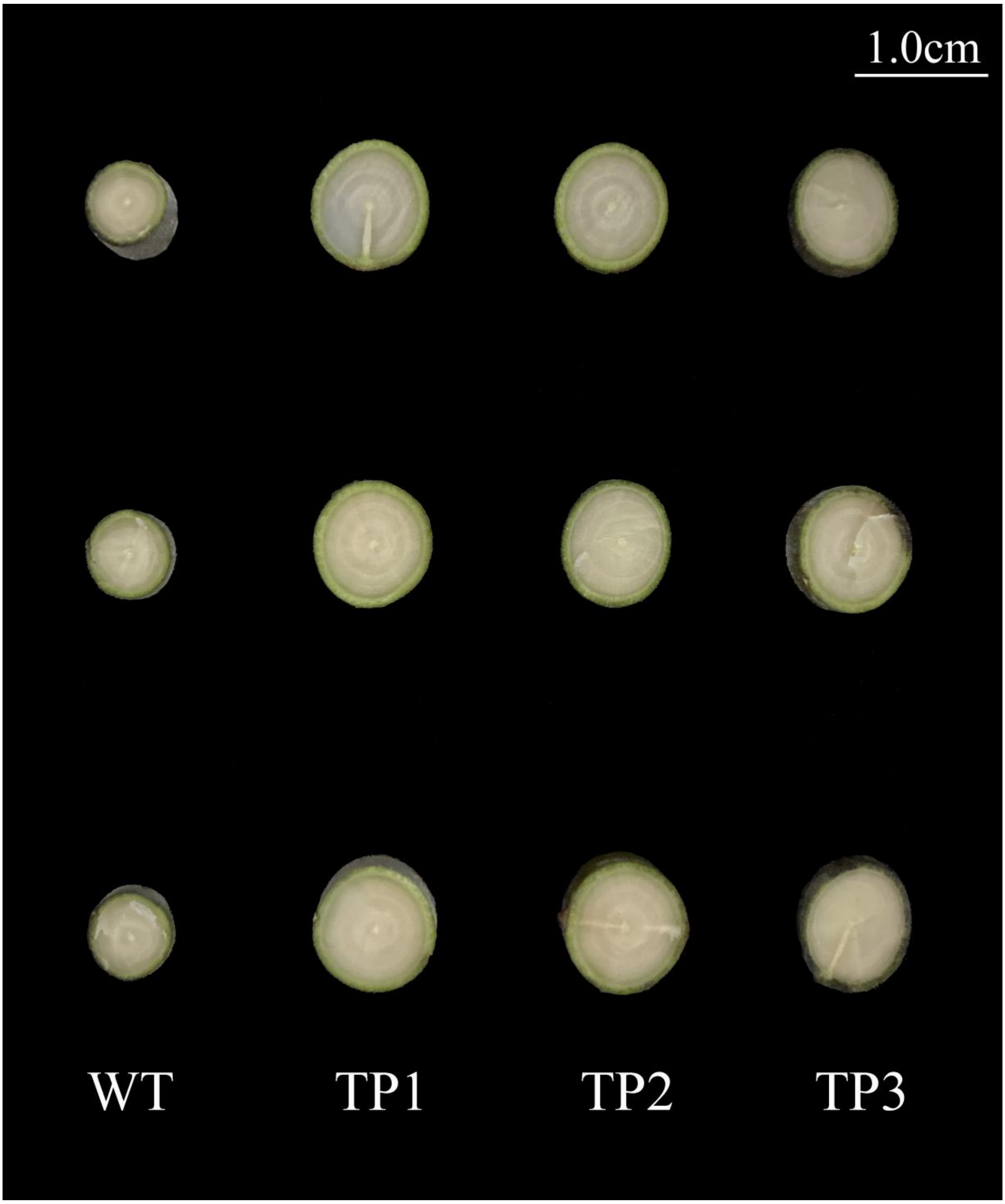

Figure S3. Cross sections of stems from the same parts of 90-d-old wild-type and transgenic plants are shown (lines 1, 2 and 3).

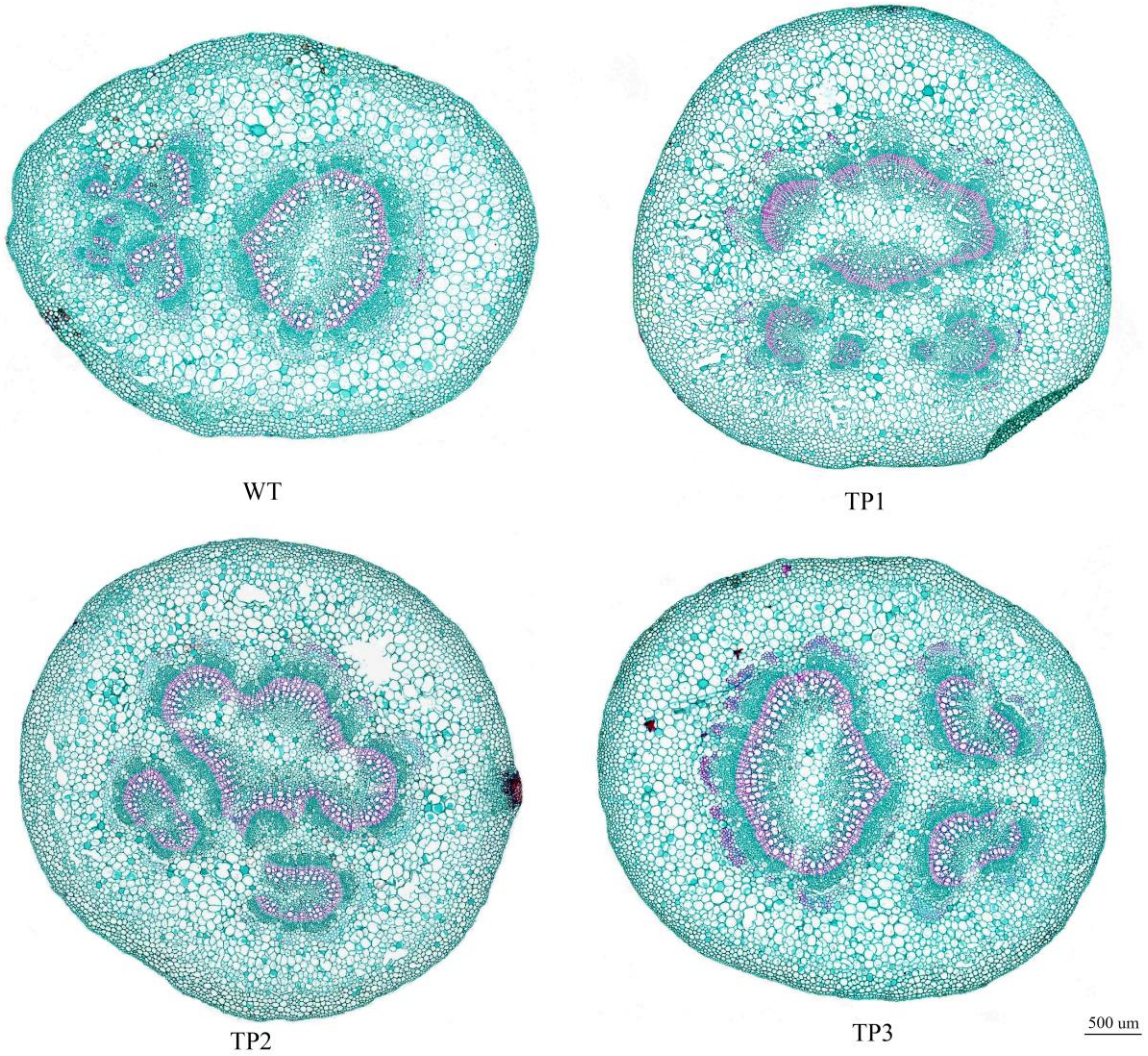

Figure S4. Morphological characteristics of complete petiole cross section of transgenic plants. A. The number of vessels and sieve tubes on the transverse section of petiole of transgenic lines per unit area. B. Number of catheters. C. Total number of conduits and sieve tubes. Error bars represent the SDs from three biological replicates. * and ** indicate significant differences in comparison to WT at $p < 0.05$ and $p < 0.01$, respectively (Student's t-test).

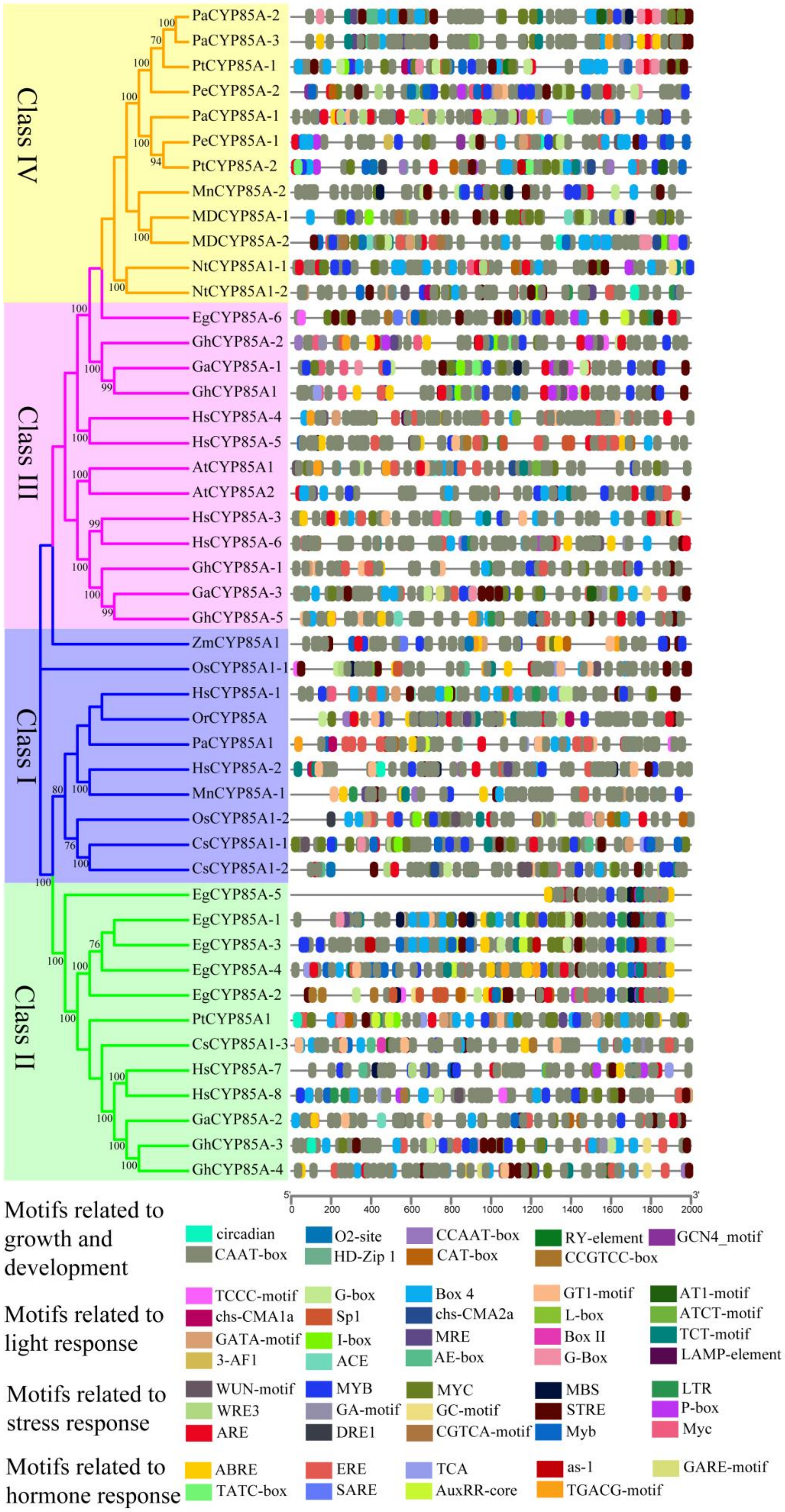
Class IV
Class III
Class I
Class II
PaCYP85A-2
PaCYP85A-3
PtCYP85A-1
PeCYP85A-2
PaCYP85A-1
PeCYP85A-1
PtCYP85A-2
MnCYP85A-2
MDCYP85A-1
MDCYP85A-2
NtCYP85A1-1
NtCYP85A1-2
EgCYP85A-6
GhCYP85A-2
GaCYP85A-1
GhCYP85A1
HsCYP85A-4
HsCYP85A-5
AtCYP85A1
AtCYP85A2
HsCYP85A-3
HsCYP85A-6
GhCYP85A-1
GaCYP85A-3
GhCYP85A-5
ZmCYP85A1
OsCYP85A1-1
HsCYP85A-1
OrCYP85A
PaCYP85A1
HsCYP85A-2
MnCYP85A-1
OsCYP85A1-2
CsCYP85A1-1
CsCYP85A1-2
EgCYP85A-5
EgCYP85A-1
EgCYP85A-3
EgCYP85A-4
EgCYP85A-2
PtCYP85A1
CsCYP85A1-3
HsCYP85A-7
HsCYP85A-8
GaCYP85A-2
GhCYP85A-3
GhCYP85A-4
5'
0 200 400 600 800 1000 1200 1400 1600 1800 2000
3'
Motifs related to growth and development
circadian
CAAT-box
O2-site
HD-Zip 1
CCAAT-box
CAT-box
RY-element
CCGTCC-box
GCN4_motif
Motifs related to light response
TCCC-motif
chs-CMA1a
GATA-motif
3-AF1
G-box
Sp1
I-box
ACE
Box 4
chs-CMA2a
MRE
AE-box
GT1-motif
L-box
Box II
G-Box
AT1-motif
ATCT-motif
TCT-motif
LAMP-element
Motifs related to stress response
WUN-motif
WRE3
ARE
MYB
GA-motif
DRE1
MYC
GC-motif
CGTCA-motif
MBS
STRE
Myb
LTR
P-box
Myc
Motifs related to hormone response
ABRE
TATC-box
ERE
SARE
TCA
AuxRR-core
as-1
TGACG-motif
GARE-motif

Figure S5. Several cis-acting elements, including light and hormone response and circadian rhythm elements, were present in the *OsCYP85A* promoter
