# Supplementary material for: The Overexpression of Oryza sativa L. CYP85A1 Promotes Growth and Biomass Production in Transgenic Trees
Source: Int J Mol Sci. 2023 Mar 30;24(7):6480. doi: 10.3390/ijms24076480 (PMC10095185; doi:10.3390/ijms24076480)
Supplement: Supplementary file 1 [file ijms-24-06480-s001.zip › Table S1.pdf]

Table S1 information of CYP85A genes

Gene ID	Gene name	Chromosome position			Protein Properties			Subcellular
		Chr	Start	End	N0.of AA	Molecular Weight (KD)	PI	
AtCYP85A1	AT5G38970.1	Chr5	15594674	15597885	465	53.77	9.08	Endoplasmic reticulum.
AtCYP85A2	AT3G30180.1	Chr3	11810737	11813765	465	53.81	8.28	Endoplasmic reticulum.
OsCYP85A1-1	LOC_Os03g40540.1	Chr3	22538341	22542520	469	52.82	9.33	Endoplasmic reticulum.
OsCYP85A1-2	LOC_Os05g01120.1	Chr5	101365	104149	518	58.61	9.46	Endoplasmic reticulum.
PeCYP85A-1	XP_011021067.1	NW_011499856.1	616166	620089	464	53.61	9.46	Endoplasmic reticulum.
PeCYP85A-2	XP_011008610.1	NW_011500351.1	77333	81043	464	53.36	9.45	Endoplasmic reticulum.
PtCYP85A-1	Potri.004G117700.2	Chr4	11124690	11128606	464	53.31	9.33	Endoplasmic reticulum.
PtCYP85A-2	Potri.017G099000.1	Chr17	11095937	11100122	464	53.62	9.46	Endoplasmic reticulum.
PtCYP85A1	Potri.010G156800.3	Chr10	16396418	16399935	465	53.24	8.96	Endoplasmic reticulum.
MDCYP85A-1	MD09G1073300	Chr09	5088511	5091448	464	53.33	8.86	Endoplasmic reticulum.
MDCYP85A-2	MD17G1064800	Chr17	5247293	5250190	464	53.25	8.75	Endoplasmic reticulum.
CsCYP85A1-1	CSS0014427.1	Chr2	15840686	15843006	374	43.16	6.84	Endoplasmic reticulum.
CsCYP85A1-2	CSS0004050.1	Chr2	15736618	15738937	374	43.24	7.18	Endoplasmic reticulum.
CsCYP85A1-3	CSS0009174.1	Chr1	204553198	204557012	465	53.62	8.89	Endoplasmic reticulum.
NaCYP85A1-1	NIATv7_g15735.t1	chr02	36772792	36776480	464	53.65	9.3	Endoplasmic reticulum.
NaCYP85A1-2	NIATv7_g23040.t1	scaffold02089	110316	113124	464	53.45	9.1	Endoplasmic reticulum.
GaCYP85A-1	XP_017618748.1	Chr13	61562150	61564733	466	53.42	9.06	Endoplasmic reticulum.
GaCYP85A-2	XP_017641393.1	Chr1	10726552	10729381	466	53.74	9.12	Endoplasmic reticulum.
GaCYP85A-3	XP_017641396.1	Chr1	10726552	10729391	465	53.65	9.12	Endoplasmic reticulum.

PaCYP85A-1	XP_034906314.1	NW_023271228.1	301974	306077	359	41.06	9.53	Endoplasmic reticulum.
PaCYP85A-2	XP_034922746.1	NW_023271569.1	750737	754412	464	53.28	9.39	Endoplasmic reticulum.
PaCYP85A1	XP_034907210.1	NW_023271243.1	390079	393270	462	52.88	8.95	Endoplasmic reticulum.
PaCYP85A-3	XP_034922744.1	NW_023271569.1	750740	754412	464	53.28	9.39	Endoplasmic reticulum.
ZmCYP85A1	Zm00001d033180_P001	Chr1	253162491	253166753	457	51.32	9.48	Endoplasmic reticulum.
OrCYP85A	orange1.1g037545m	scaffold00019	1549676	1551731	383	44.72	8.83	Endoplasmic reticulum.
EgCYP85A-1	Eucgr.L01750.1	scaffold_335	5513	8250	466	53.63	9.29	Endoplasmic reticulum.
EgCYP85A-2	Eucgr.J01402.1	Chr10	16755390	16758433	466	53.64	9.3	Endoplasmic reticulum.
EgCYP85A-3	Eucgr.J01404.1	Chr10	16765706	16768470	470	54.11	9.35	Endoplasmic reticulum.
EgCYP85A-4	Eucgr.J01407.1	Chr10	16784348	16787097	470	54.08	9.35	Endoplasmic reticulum.
EgCYP85A-5	Eucgr.L03131.1	scaffold_2072	717	3462	417	48.38	9.42	Endoplasmic reticulum.
EgCYP85A-6	Eucgr.I02054.1	Chr09	30327565	30331108	465	53.36	9.23	Endoplasmic reticulum.
GhCYP85A-1	Gohir.D07G187300.1	Chr7	47641367	47644731	465	53.73	9.19	Endoplasmic reticulum.
GhCYP85A1	Gohir.A13G084216.1	Chr13	29324000	29326434	466	53.43	9.06	Endoplasmic reticulum.
GhCYP85A-2	Gohir.D13G083600.1	Chr13	16622204	16624731	466	53.57	9.09	Endoplasmic reticulum.
GhCYP85A-3	Gohir.A07G079400.1	Chr7	12229846	12233418	466	53.64	9.31	Endoplasmic reticulum.
GhCYP85A-4	Gohir.D07G084700.1	Chr7	10372158	10375698	466	53.69	9.17	Endoplasmic reticulum.
GhCYP85A-5	Gohir.A07G180600.2	Chr7	81864892	81867776	465	53.68	9.12	Endoplasmic reticulum.
MnCYP85A-1	XP_024018327.1	NW_010358777.1	353822	359037	468	54.16	8.78	Endoplasmic reticulum.
MnCYP85A-2	XP_010099314.1	NW_010362042.1	377423	381013	465	53.66	8.97	Endoplasmic reticulum.
HsCYP85A-1	XP_039034793.1	NW_024063186.1	2388860	2391905	485	56.53	7.68	Endoplasmic reticulum.
HsCYP85A-2	XP_039034687.1	NW_024063182.1	70302	72645	469	53.88	9.21	Endoplasmic reticulum.

HsCYP85A-3	XP_039000992.1	NW_024062747.1	1631590	1635783	469	54.00	9.03	Endoplasmic reticulum.
HsCYP85A-4	XP_039029807.1	NW_024061957.1	243119	245618	466	53.46	9.18	Endoplasmic reticulum.
HsCYP85A-5	XP_039021115.1	NW_024062984.1	111852	114284	466	53.40	9.3	Endoplasmic reticulum.
HsCYP85A-6	XP_039003683.1	NW_024062775.1	5252940	5255597	465	53.45	9.12	Endoplasmic reticulum.
HsCYP85A-7	XP_038990963.1	NW_024062631.1	50330	53003	465	53.69	9.01	Endoplasmic reticulum.
HsCYP85A-8	XP_039021620.1	NW_024062987.1	972589	975380	465	53.81	8.62	Endoplasmic reticulum.
