# Supplementary figures and images for: The Overexpression of Oryza sativa L. CYP85A1 Promotes Growth and Biomass Production in Transgenic Trees
Source: Int J Mol Sci. 2023 Mar 30;24(7):6480. doi: 10.3390/ijms24076480 (PMC10095185; doi:10.3390/ijms24076480)

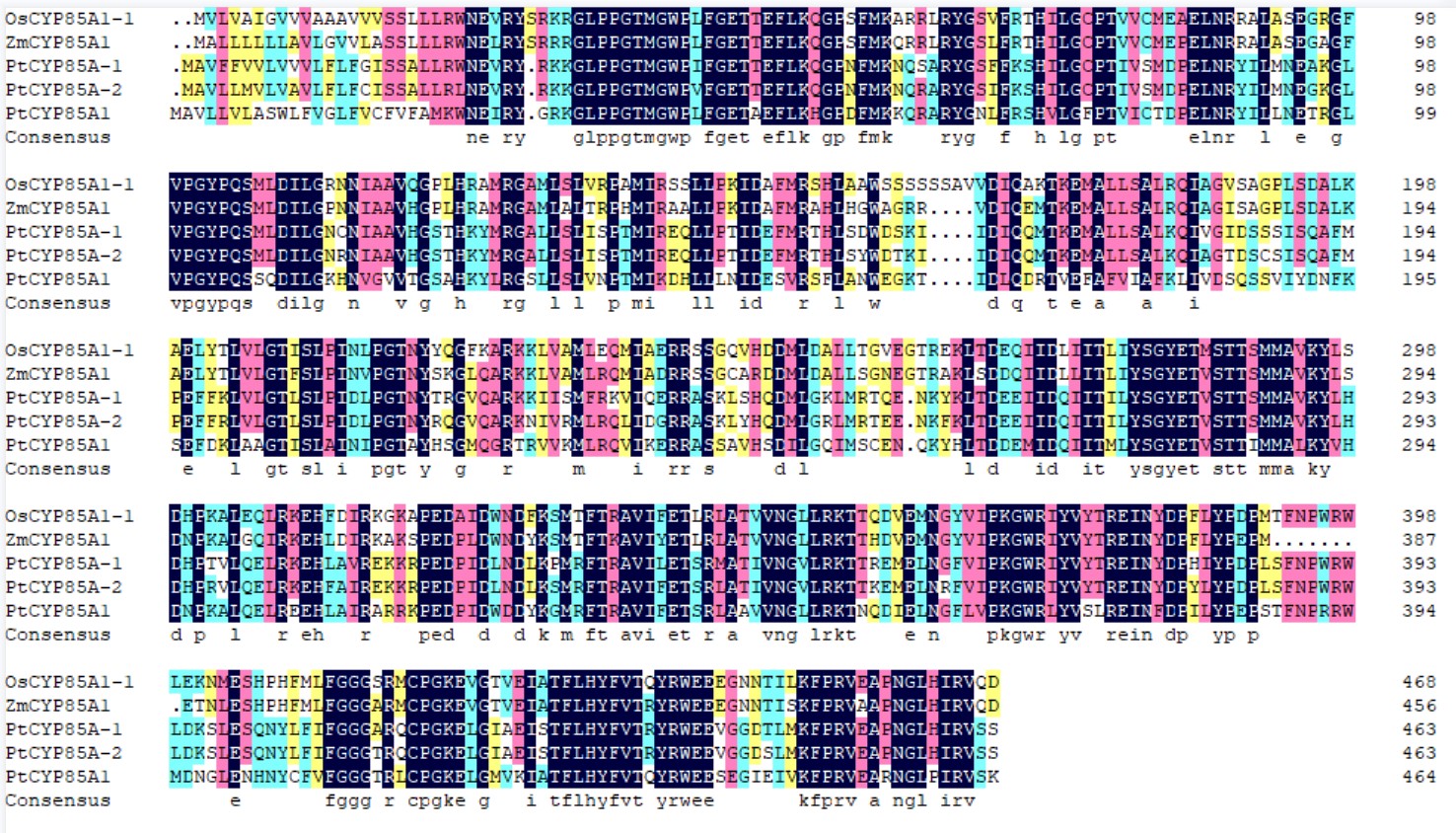

Supplement: Supplementary file 1 [file ijms-24-06480-s001.zip › Fig. S1.jpg]

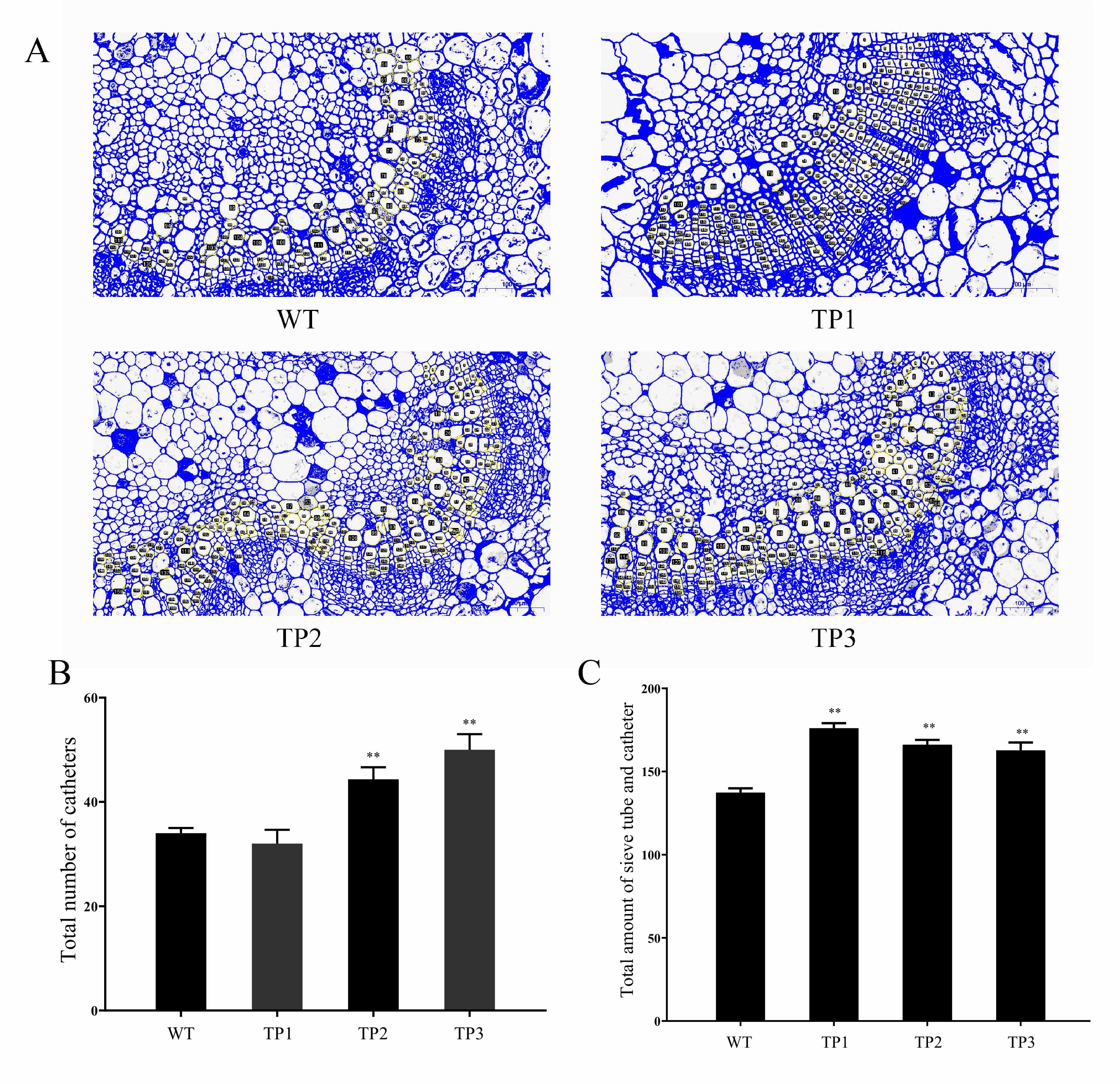

Supplement: Supplementary file 1 [file ijms-24-06480-s001.zip › Fig. S2.jpg]

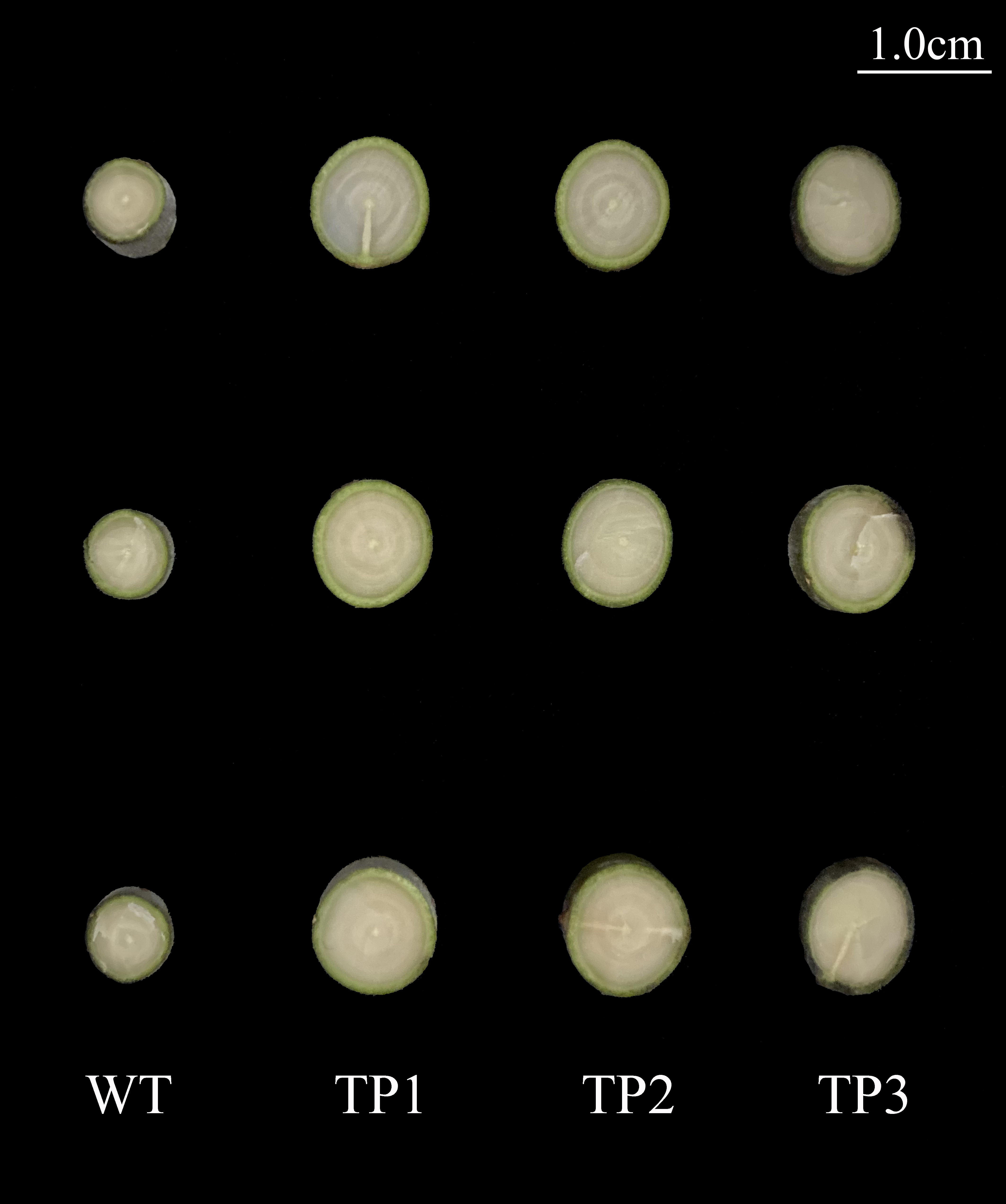

Supplement: Supplementary file 1 [file ijms-24-06480-s001.zip › Fig. S3.jpg]

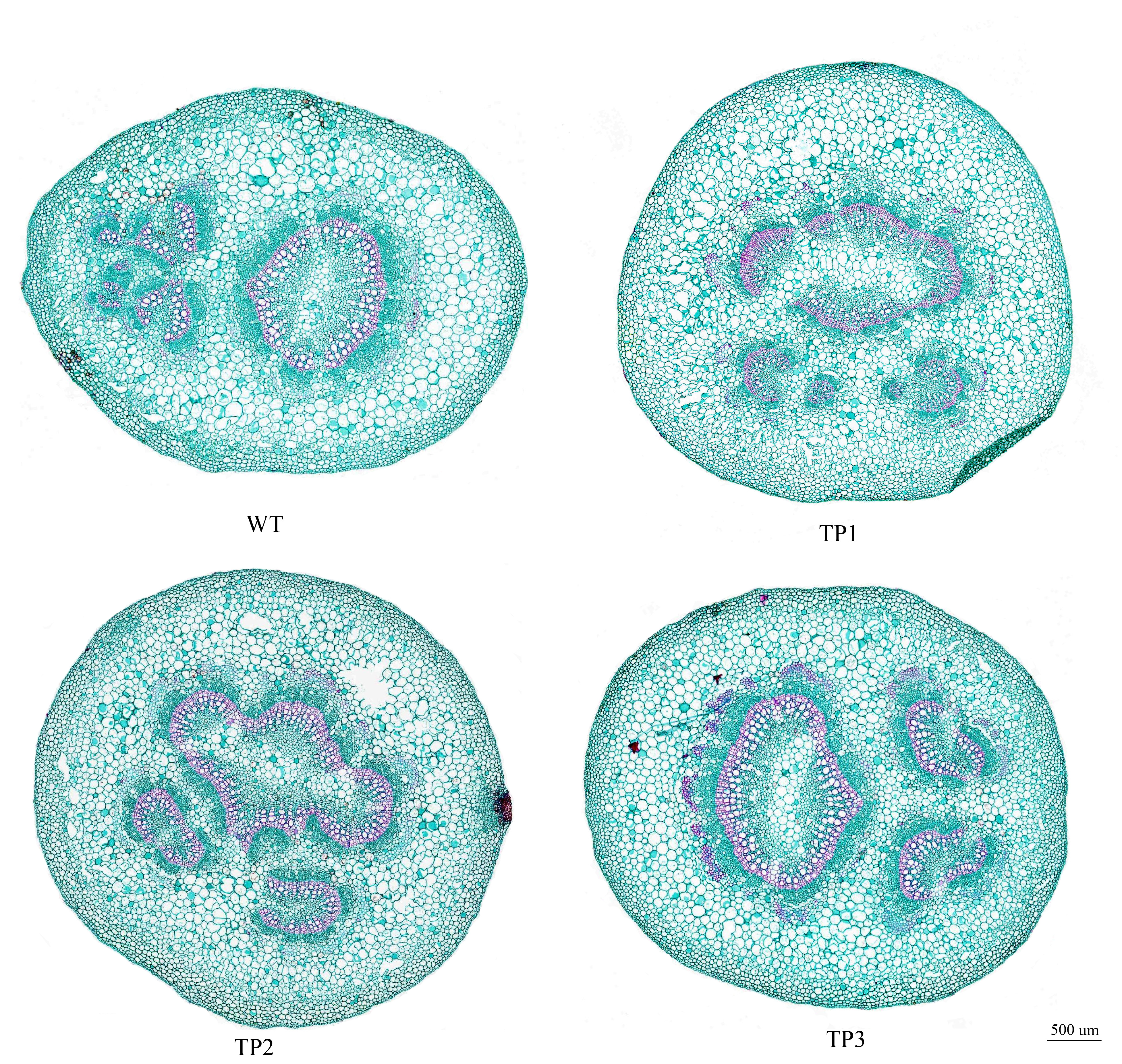

Supplement: Supplementary file 1 [file ijms-24-06480-s001.zip › Fig. S4.jpg]

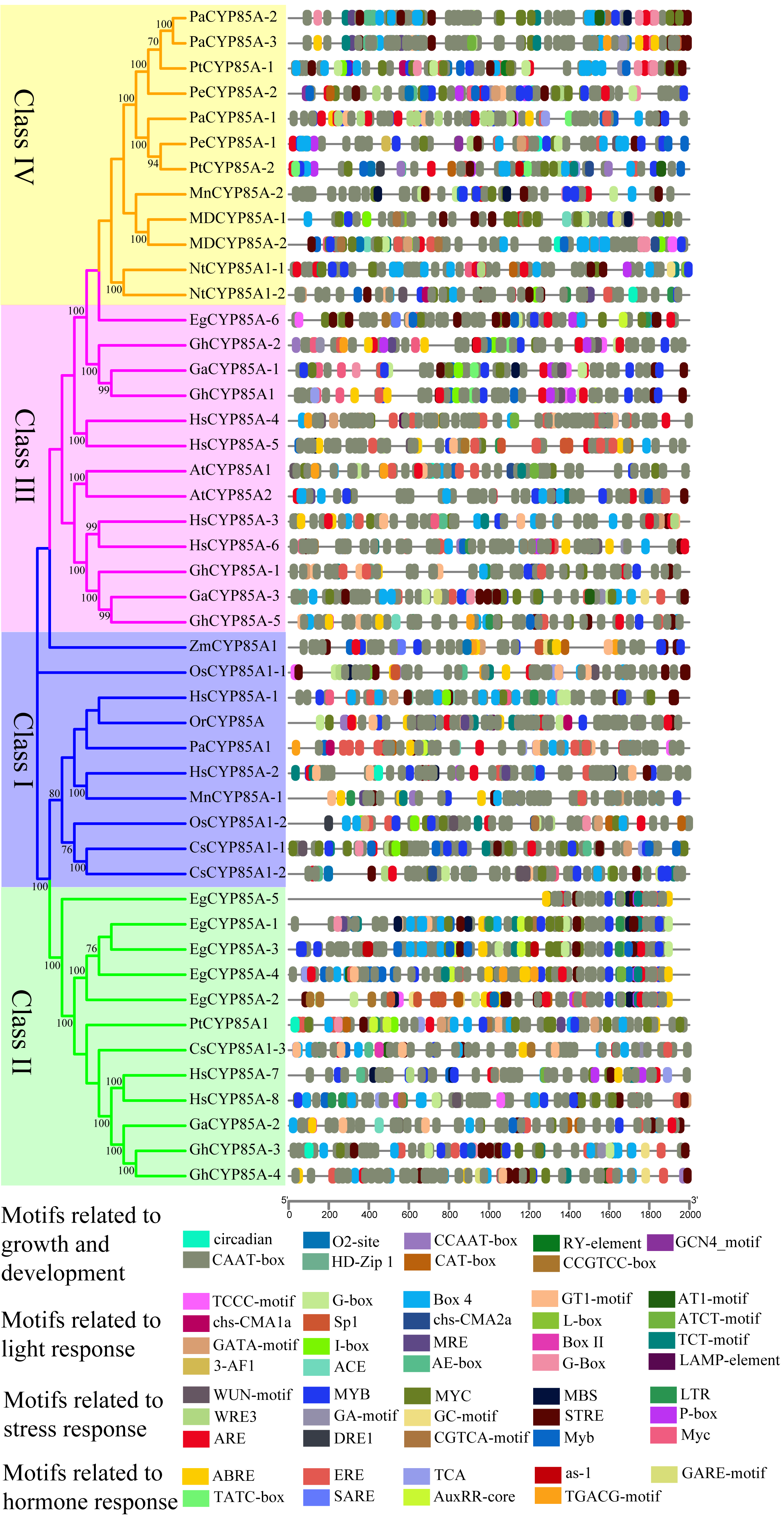

Supplement: Supplementary file 1 [file ijms-24-06480-s001.zip › Fig. S5.tif]
